# Ginseng Bioactive Components as Gut-Brain Axis-Targeted Modulators: Therapeutic Potential and Mechanisms in Multifactorial Diseases

**DOI:** 10.3390/nu18111778

**Published:** 2026-05-31

**Authors:** Silu Liu, Lanshi Tian, Weijia Chen, Jianan Geng, Zhongmei He, Jia Zhou

**Affiliations:** 1 College of Chinese Medicinal Materials, Jilin Agricultural University, Changchun 130118, China; liusilu0616@163.com (S.L.); 18205037129@163.com (L.T.); chenweijia_jlau@163.com (W.C.); gengjianan@jlau.edu.cn (J.G.); 2 College of Pharmacy, Yanbian University, 997 Park Road, Yanji 133002, China

**Keywords:** gut-brain axis (GBA), ginseng, ginsenosides, ginseng polysaccharides, gut microbiota

## Abstract

Ginseng (*Panax ginseng* C. A. Mey.) is a classic traditional Chinese herbal medicine with a history of clinical use that spans millennia. Its utilisation has long been established in the regulation of physical and mental equilibrium, in addition to the amelioration of conditions pertaining to the heart, spleen, and brain. Recent studies have indicated that the core biological activity of the substance under investigation is mediated by key active components such as ginsenosides, polysaccharides, and polyphenols. These components are closely associated with the regulation of the gut-brain axis (GBA). However, extant reviews have predominantly concentrated on individual diseases or specific mechanisms, thereby lacking a thorough investigation into the comprehensive analysis of how ginseng components exert systemic effects via the GBA. This review systematically searched and analyzed published studies in major databases regarding the regulation of the GBA by ginseng bioactive components, summarizing the latest advances in its role as a multifactorial disease intervention regulator targeting the GBA. It has been demonstrated that ginseng components exert a multifaceted GBA regulatory effect through interconnected mechanisms, including modulation of the gut microbiota, protection of the intestinal barrier, anti-inflammatory actions, and regulation of neurotransmitters, showing promising preclinical therapeutic potential in neurodegenerative diseases, mood disorders, metabolic diseases, and gastrointestinal disorders. Contrary to previous reviews, which focused on the description of individual ginseng components or specific diseases, this study provides a comprehensive analysis of how various bioactive components of ginseng modulate the gut-brain axis in relation to multiple disease categories through a systematic review. However, the preponderance of extant evidence derives from preclinical studies and necessitates further validation through clinical trials. This review provides pivotal directions and theoretical underpinnings for the clinical translation of ginseng’s bioactive components and the development of disease intervention strategies targeting the gut-brain axis.

## 1. Introduction

In recent years, the concept of the gut-brain axis (GBA) has emerged as a central area of research. The GBA facilitates bidirectional regulation between the gut and the brain through neural, immune, endocrine, and metabolic pathways, and involves complex interactions among these pathways. It also plays a central role in neurodegenerative diseases [1], psychiatric disorders [2], and gastrointestinal diseases.

The gut microbiota is defined as a community of trillions of microorganisms residing within the gastrointestinal tract, thereby serving as a crucial link connecting the GBA. It is estimated that there are myriad microorganisms that partake in a variety of physiological processes within the human body. These microorganisms are responsible for the production of numerous metabolites, including short-chain fatty acids (SCFAs) and neurotransmitters. These metabolites are produced during various stages of digestion, nutrient absorption, and immune system regulation. These metabolites have the capacity to exert a further influence on intestinal function and immune responses. These substances have the potential to traverse the blood-brain barrier (BBB) or exert their effects on the enteric nervous system, facilitating the transmission of signals via the vagus nerve to the central nervous system. This process leads to a direct impact on neural signaling in the brain [3]. Dysregulation of the gut-brain axis plays a pivotal role in the pathogenesis of multifactorial diseases, with gut-brain axis disruption increasing intestinal permeability, triggering systemic inflammatory responses, and causing neurotransmitter metabolic abnormalities. Moreover, neurodegenerative diseases such as Alzheimer’s disease (AD) and Parkinson’s disease (PD) have been linked to dysfunction of the gut-brain axis. Growing evidence indicates that toxins or inflammatory mediators produced by gut microbiota can excessively activate microglia in the brain, thereby initiating or exacerbating neurodegeneration, ultimately leading to chronic neuroinflammation and neuronal damage [4].

*Panax ginseng*, a herb with a long history of use in traditional Chinese medicine, contains multiple bioactive components and has been found to possess anti-inflammatory [5], antioxidant [6], neuroprotective [7], and gut microbiota-modulating functions [8]. Recent studies indicate that it may have therapeutic potential in multifactorial diseases by regulating the gut-brain axis [8,9]. The gut-brain axis, a core regulatory system within the body, has been demonstrated to be closely associated with the onset and progression of various multifactorial diseases. The bioactive components of ginseng, which act as modulators of the gut-brain axis, demonstrate significant potential. This provides novel insights and potential strategies for the treatment and intervention of these complex diseases.

A number of recent reviews have explored the relationship between ginseng and the gut-brain axis; however, the majority of these focus exclusively on a single component (primarily ginsenosides) or specific disease categories. A conspicuous absence in the extant literature is a comprehensive review that systematically integrates the various major bioactive components of ginseng, their regulation of the gut-brain axis, and the mechanisms by which these components modulate the gut-brain axis to influence multiple major diseases. The objective of this study is to address this knowledge gap by conducting a comprehensive review of the various active components in ginseng. This review will explore how these components modulate the gut-brain axis to treat various diseases. Additionally, the study will evaluate the translational potential and limitations of current research.

## 2. Methods

**Figure 1:**
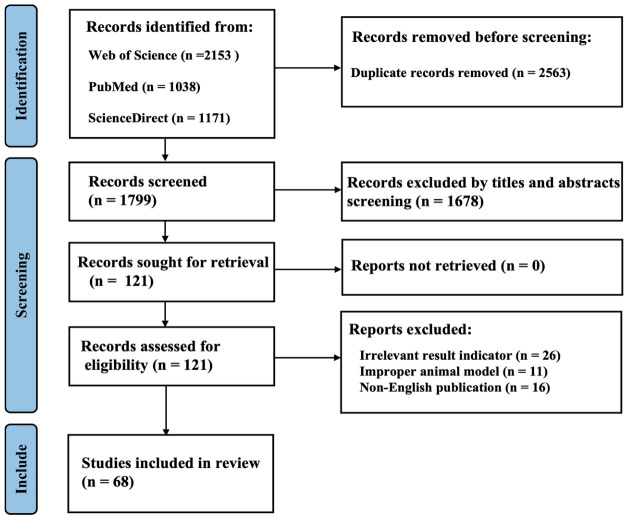
Flowchart of the literature screening process.

This systematic review strictly adhered to the PRISMA (Preferred Reporting Items for Systematic Reviews and Meta-Analyses) guidelines [10]. A comprehensive literature search was conducted in the Web of Science, PubMed, and ScienceDirect databases to collect relevant studies published from the inception of each database through September 2025. Search terms included “active components of ginseng,” “ginsenosides,” “ginseng polysaccharides,” “gut microbiota,” “gut-brain axis,” and combinations of these terms. Studies were included that investigated the modulatory effects of ginseng and its major components on the gut-brain axis and their potential mechanisms in mammalian in vivo or in vitro models. Exclusion criteria included studies unrelated to gut-brain axis modulation, non-mammalian models, review articles, conference abstracts, duplicate publications, and retracted papers. After removing duplicate records, eligible studies were initially identified by screening titles and abstracts, followed by full-text evaluation of potentially relevant records. The specific screening process is illustrated in Figure 1.

## 3. Concept of Gut-Brain Axis

The gut-brain axis refers to the bidirectional communication system formed between the gastrointestinal system and the brain through neural, endocrine, and immune mechanisms. The integumentary system comprises the gastrointestinal tract, the central nervous system, the autonomic nervous system, the enteric nervous system, the endocrine system, and the immune system. The components interact and influence each other, thereby collectively maintaining the body’s physiological and psychological equilibrium [11,12]. Within this intricate network, the gut and brain exchange information to regulate diverse physiological functions. For instance, changes in gut physiology can travel via the gut-brain axis to influence brain function, while emotional states and stress signals from the brain can similarly affect intestinal motility, secretion, and absorption [13]. The gut-brain axis, as a complex bidirectional communication system linking the gastrointestinal tract and the brain, plays a crucial role in maintaining physiological balance and health. The communication process encompasses multiple levels, including neural, endocrine, and immune pathways, with the gut microbiota playing a pivotal role.

### 3.1. Components

#### 3.1.1. Neural Pathways

The gut-brain axis comprises the central nervous system (CNS), the enteric nervous system (ENS), and the autonomic nervous system (ANS). The CNS is responsible for regulating higher neural activities and integrating information. In contrast, the ENS is often referred to as the body’s “second brain” and contains tens of thousands of neurons that are extensively distributed within the intestinal wall. It has been demonstrated to independently regulate fundamental intestinal functions, including motility, secretion, and blood flow, without the need for intervention from the CNS [14]. The autonomic nervous system modulates intestinal activity through the sympathetic and parasympathetic nervous systems, thereby maintaining a state of functional equilibrium [15]. It has been established that neural pathways function as the primary conduit for information transmission within the gut-brain axis. It has been demonstrated that neurons within the gastrointestinal system and the vagus nerve, which modulates intestinal nerves, can directly interact with microbial products and microbiota-dependent immune mediators [16]. Local signals are transmitted via sensory circuits to brain regions that have been shown to be associated with cognition, emotion, and somatosensation. Conversely, the brain exerts a regulatory influence over gastrointestinal homeostasis through the release of efferent projections from the vagus nerve and the spinal cord that target the intestinal mucosa. These projections interact with the enteric nervous system, contributing to the maintenance of internal environment stability. Through top-down signaling and the modulation of gut neuronal activity by microbiota-associated molecules, which in turn affects gastrointestinal physiology, local immune function, and gut microbiota composition [17]. When the gastrointestinal system is exposed to noxious stimuli, enteric neurons initiate the transmission of signals to the brain via the vagus nerve. The brain receives these signals and generates corresponding feedback, thereby regulating intestinal motility to rapidly expel harmful substances [18].

#### 3.1.2. Endocrine Pathway

Endocrine pathways have also been demonstrated to play a crucial role in gut-brain axis communication. The transmission of signals through enteroendocrine cells by microbial products and metabolites has been demonstrated to regulate neurotransmitter secretion [19]. Furthermore, certain subpopulations within the gut microbiota can directly synthesize and release neurotransmitters. These substances have the potential to be absorbed into the portal venous system, which may allow them to traverse the BBB and directly influence central neuronal activity [20]. The gut microbiota and hypothalamic-pituitary-adrenal cortex axis (HPA axis) have been demonstrated to influence neuroendocrine signaling pathways in a mutually reinforcing manner. Stress-induced activation of the HPA axis has been demonstrated to affect gastrointestinal function, altering the composition of the gut microbiota. Conversely, reduced microbiota abundance modifies HPA axis function. Research indicates that during periods of stress, the HPA axis is activated, leading to increased cortisol and other hormone secretion, affecting intestinal motility and digestive fluid secretion while altering gut microbiota structure. Conversely, dysbiosis has been demonstrated to influence the HPA axis, thereby modulating hormone secretion levels and consequently, systemic stress responses [21].

#### 3.1.3. Immune Pathway

The immune pathway is pivotal for communication along the gut-brain axis, playing a crucial role in maintaining immune homeostasis and neural function. Microbial metabolites, including SCFAs, and membrane components can shape immune homeostasis and modulate pro-inflammatory or anti-inflammatory states in local immune responses [22,23]. Furthermore, the gut microbiota participates in regulating the development and function of microglia [24], the resident immune cells of the brain that maintain cerebral immune balance and neural function. During neuroinflammatory episodes, increased BBB permeability allows microbial products and peripheral immune cells to rapidly infiltrate the brain parenchyma, thereby initiating or exacerbating neuroinflammatory responses [25]. Conversely, brain inflammation disrupts gut microbiota homeostasis, further intensifying neuroimmune responses and worsening pathology [26], thereby creating a bidirectional inflammatory vicious cycle.

#### 3.1.4. Main Participating Parts

The gut microbiota plays a pivotal role in the gut-brain axis, typically comprising a vast and diverse array of microorganisms, including bacteria, fungi, and viruses. These microorganisms are involved in the process of food digestion and the absorption of nutrients [27]. Additionally, they are responsible for synthesizing beneficial substances, such as SCFAs [28]. Alterations in the composition and function of the gut microbiota are closely associated with the onset and progression of numerous major diseases, including irritable bowel syndrome [29], inflammatory bowel disease [30], and neurological disorders [31]. The discovery that the composition of the gut microbiota in PD differs significantly from that of healthy individuals suggests a potential influence of altered bacterial species on neuroinflammation and neurotransmitter metabolism via the gut-brain axis. This phenomenon may contribute to the pathogenesis of PD [32].

The vagus nerve serves as a pivotal structure in the gut-brain axis neural pathway, functioning as a conduit for bidirectional communication between the nervous and gastrointestinal systems. The vagus nerve is the longest and most widely distributed cranial nerve in the human body. The vast majority of its nerve fibers originate in the gut and project to the brain. Changes in gut microbiota can influence brain activity via the vagus nerve [33], while conversely, brain signals can regulate intestinal physiological functions through the vagus nerve [34].

Neurotransmitters are crucial chemical messengers in the gut-brain axis, with many, such as serotonin 5-hydroxytryptamine (5-HT), gamma-aminobutyric acid (GABA), and dopamine, synthesized in both the brain and gut [35]. Over 90% of serotonin is produced in the gut, regulating intestinal function and influencing mood and cognition via systemic circulation or the vagus nerve [36,37]. Gut microbiota also modulate GABA and dopamine metabolism to affect central nervous system activity [38,39].

Inflammatory cytokines (e.g., TNF-α) mediate bidirectional immune communication along the gut-brain axis [40]. Intestinal inflammation-induced cytokine release activates central microglia and disrupts neuronal function, while brain inflammation in turn alters gut microbiota homeostasis [41,42]. In patients with inflammatory bowel disease, persistent intestinal inflammation promotes massive release of inflammatory cytokines. Once these enter the bloodstream, they can impair brain neural function, leading to psychiatric symptoms like anxiety and depression [43]. Conversely, abnormal emotional states in the brain may also exacerbate intestinal inflammation through the gut-brain axis [44].

**Figure 2:**
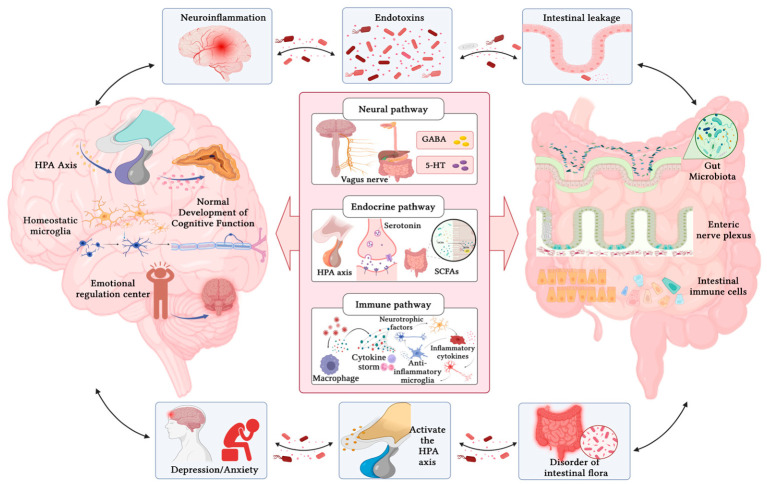
Bidirectional communication mechanisms of the gut-brain axis.

Consequently, as a multifaceted and exacting bidirectional communication system, the neural, endocrine, and immune pathways of the gut-brain axis are intricately interwoven. It has been established that a multitude of participants, including the intestinal microbiota, the vagus nerve, neurotransmitters, and inflammatory cytokines, collaborate to preserve the physiological and psychological equilibrium of the body. It has been demonstrated that the gut-brain axis is of great significance for revealing the pathogenesis of multifactorial diseases and in the development of novel treatment strategies (Figure 2).

## 4. Pharmacological Properties of the Active Ingredients of Ginseng

### 4.1. Ginsenoside Components

Ginsenosides, as the primary active constituents of ginseng, are a class of glycoside compounds featuring a tetraterpenoid dammarane or pentaterpenoid oleanane skeleton. Based on differences in aglycone structure, they can be classified into protopanaxadiol-type (PPD-type, e.g., Rb1, Rb2, Rc, Rd, Rg3, CK), protopanaxatriol-type (PPT-type, e.g., Re, Rg1, Rg2, Rh1), and oleanane-type (Ro). Accumulating preclinical studies suggest that ginsenosides may exert bidirectional regulatory effects on the gut-brain axis through their gut microbiota metabolites (e.g., compound CK), involving mechanisms such as HPA axis modulation, neuroinflammation suppression, and intestinal barrier repair [45]. Regarding neuromodulation, ginsenoside Rg1 has been shown to mitigate cerebral ischemic injury by regulating the miR-144/Nrf2-ARE pathway [46], while Rb1 protects neurons in depression by inhibiting mitochondrial autophagy-mediated astrocytic pyroptosis [47]. Recent research reveals that ginsenoside Ro significantly reduces Aβ deposition, microglial activation, and proinflammatory cytokine release in APP/PS1 Alzheimer’s disease model mice by inhibiting the IBA1/GFAP-MAPK signaling pathway, while simultaneously improving cognitive impairment and anxiety-related behaviors [48]. Furthermore, regarding HPA axis regulation, ginsenosides significantly reversed abnormal hippocampal cortisol levels in post-traumatic stress disorder (PTSD) model rats, elevated 5-HT concentrations, and reduced neuronal apoptosis rates, confirming their neuroprotective effects through HPA axis homeostasis regulation [49,50].

Ginsenosides have demonstrated a multi-target repair mechanism in protecting the intestinal barrier in preclinical models. Research confirms that Ginseng total saponins (GTSs) significantly enhance the expression of tight junction proteins occludin and claudin-1 in simulated microgravity stress models, increase goblet cell numbers and mucus secretion, and promote the production of beneficial metabolites such as propionic acid and butyric acid while reshaping the gut microbiota structure [51]. Notably, the rare ginsenoside Rh4 effectively counteracts cisplatin chemotherapy-induced intestinal mucosal barrier damage by activating mitochondrial autophagy. This process restores intestinal villus structural integrity and suppresses apoptosis through upregulating tight junction protein expression [52]. Furthermore, the ginsenoside metabolite CK has been shown to selectively inhibit the PHD2 protein, modulate collagen hydroxymethylation, diminish aberrant platelet-endothelial adhesion, and enhance microcirculatory dysfunction within the body [53].

The regulation of metabolites by the microbiome constitutes the primary mechanism through which ginsenosides modulate the gut-brain axis. Ginsenosides undergo a gradual process of deglycosylation, resulting in the formation of more bioavailable secondary metabolites. This process is facilitated by β-glucosidase, a carbohydrate-active enzyme secreted by gut microbiota [54]. Furthermore, ginsenosides have been shown to regulate bile acid metabolism [55]. Research indicates that ginsenoside Rh4 enhances 7α-hydroxysteroid dehydrogenase activity by enriching Akkermansia species, thereby promoting ursodeoxycholic acid (UDCA) production. This activates the FXR receptor and inhibits the TLR4-NF-κB inflammatory pathway [56]. Thus, ginsenosides achieve bidirectional regulation of the gut-brain axis through multidimensional interactions within the “microbiome-metabolism-immune-neuro” network. These findings offer novel insights for developing innovative therapeutic strategies targeting multifactorial diseases.

In summary, ginsenosides act as stable modulators of GBA, with consistent results observed across various animal disease models, including those for Alzheimer’s disease, Parkinson’s disease, depression, and colitis. Fecal microbiota transplantation experiments further confirm the causal role of gut microbiota remodelling in mediating these effects. In vitro studies have also elucidated the potential molecular mechanisms underlying tight junction protection and anti-inflammatory effects. However, most preclinical studies have used purified ginsenosides at doses far exceeding physiological levels. Furthermore, the low oral bioavailability of ginsenoside-rich formulations remains a significant barrier to clinical translation.

### 4.2. Ginseng Polysaccharide Components

Ginseng polysaccharides represent a pivotal category of bioactive macromolecules in ginseng, along with saponins. These polysaccharides encompass a diverse array of types, including arabinogalactans, rhamnogalacturans, and homopolysaccharides. Their molecular weights range from tens to hundreds, and this structural diversity directly influences their biological activities and mechanisms of action [57]. Recent preclinical studies have revealed that ginseng polysaccharides may play a central role in alleviating neuroinflammation [58], suppressing intestinal injury [59], and regulating the microbiota [60] through their unique “microbiota-immune-metabolic” regulatory network. In neuroprotection, a 4.7-kDa ginseng-derived polysaccharide (GP4) demonstrated significant neuroprotective effects in cross-species AD models. As indicated by the extant literature, GP4 has been shown to inhibit Aβ aggregation and reduce neuroinflammation by activating mitochondrial autophagy to clear damaged mitochondria. This, in turn, has been demonstrated to improve memory deficits [61]. In addition, ginseng polysaccharides enhance mitochondrial function and exert a protective effect on H9c2 cardiomyocytes subjected to hypoxia/reoxygenation injury [62], suggesting the potential for multiorgan anti-inflammatory mechanisms.

Ginseng polysaccharides also exhibit multi-level regulatory functions in modulating the gut microbiota. A pioneering study has demonstrated that ginseng neutral polysaccharides (GPN) enhance tryptophan production by selectively enriching tryptophan-producing bacteria. This results in the activation of the aryl hydrocarbon receptor signaling pathway, upregulates intestinal stem cell marker expression, and tight junction protein expression. Fecal microbiota transplantation experiments validated that GPN can repair intestinal barrier defects in aged mice and suppress systemic inflammation [63]. Concurrently, ginseng polysaccharides demonstrated comparable microbiota-modulating effects in aquatic animal models. In a high-cotton meal diet-induced intestinal dysfunction model, ginseng polysaccharides reversed growth retardation and intestinal barrier damage by balancing beneficial-to-pathogenic bacteria ratios while synergistically activating the Nrf2/Keap1 antioxidant pathway and suppressing NF-κB/TLR inflammatory signaling [64]. In the context of spotted catfish, the dietary supplementation of ginseng polysaccharides has been shown to promote the proliferation of beneficial bacterial colonies within the gastrointestinal tract. This, in turn, has led to an enhancement in the activity of digestive enzymes and an increase in the expression of tight junction proteins. The outcomes of this process include improvements in growth performance, a strengthening of intestinal health, a modulation of immune function, and an elevation in resistance to bacterial infections [65].

It is noteworthy that structural characteristics of ginseng polysaccharides are closely associated with their functional effects. In an antibiotic-associated diarrhea (AAD) mouse model, the ginseng polysaccharides (WGP) altered the composition and diversity of the gut microbiota in AAD-affected mice, restored intestinal flora, balanced metabolic processes, and promoted mucosal recovery [66]. Furthermore, a colon-targeted microsphere system derived from ginseng polysaccharides has been shown to alleviate mucosal damage in ulcerative colitis (UC) models through a dual anti-inflammatory and microbiota-modulating mechanism [67]. These preclinical findings confirm that ginseng polysaccharides mediate bidirectional gut-brain communication via the “microbiota-metabolite-immune” axis and the “microbiota-barrier-nerve” axis.

In rodent models, ginseng polysaccharides have demonstrated strong prebiotic and gut barrier-protective activities. FMT studies have confirmed that the beneficial effects of ginseng polysaccharides are mediated through the regulation of the gut microbiota. However, the current evidence for ginseng polysaccharides is derived solely from preclinical studies, and there are no human intervention trials to confirm their effects on the gut-brain axis. Additionally, the structural heterogeneity of ginseng polysaccharides and variations in extraction methods may contribute to discrepancies in research findings.

### 4.3. Ginseng Polyphenols and Essential Oils

Ginseng polyphenols represent a pivotal class of bioactive compounds in ginseng, primarily comprising gallic acid, catechins, proanthocyanidins, and ginseng flavonoids. The abundant phenolic hydroxyl groups present in their molecular structures serve as the fundamental basis for their diverse pharmacological activities [68]. The content of volatile oil components in ginseng is lower than that of ginsenosides, which are primarily composed of terpenes and their derivatives [69]. The unique chemical structures of ginsenosides also confer specific biological functions.

Current preclinical research confirms that ginseng volatile oils can improve mitochondrial structural damage in aging models, enhance the activity of mitochondrial respiratory chain complexes, reduce the accumulation of oxidative stress products, and thereby exert effective antioxidant protective effects [70]. Notably, β-phellandrene, a key component in ginseng volatile oil, can cross the blood-brain barrier to directly act on the central nervous system. It alleviates anxiety-like behaviors by modulating GABAergic neurotransmission [71]. Concurrently, polyphenolic components have been shown to improve intestinal mucosal damage, upregulate tight junction protein expression in intestinal epithelial cells, and enhance intestinal barrier integrity to reduce intestinal permeability [72]. The synergistic effects of volatile oils and polyphenols are equally significant in the regulation of the gut-brain axis. It has been demonstrated that β-caryophyllene, a constituent of volatile oils, functions as an aromatic receptor ligand. This activation of TRPV1 channels in intestinal chromaffin cells has been shown to promote 5-HT synthesis [73]. Meanwhile, polyphenolic metabolites further induce regulatory T cell differentiation and IL-22 secretion, thereby strengthening the intestinal barrier and reducing systemic inflammation [74,75]. Consequently, this dual receptor mechanism not only effectively mitigates intestinal inflammation but also transmits signals via the vagus nerve to the nucleus of the solitary tract, thereby enhancing the plasticity of synaptic connections in the hippocampus and enhancing cognitive function.

### 4.4. Rationale for Targeting GBA

The active components of ginseng can achieve targeted intervention for multisystemic diseases through four major regulatory pathways of the gut-brain axis: neural, immune, endocrine, and microbiome-metabolism. The fundamental mechanism of this process involves the establishment of a regulatory network that functions in a synergistic manner to achieve three primary objectives: the protection of the intestinal barrier, the suppression of neuroinflammation, and the restoration of brain function. Saponins, the core active compounds, simultaneously enhance the physical barrier by upregulating tight junction proteins in the intestinal epithelium, thereby reducing endotoxin leakage into the bloodstream and lowering systemic inflammatory burden [76]. In addition, saponins have been observed to cross the blood-brain barrier, where they act on the central nervous system, inhibiting microglial activation [77] and alleviating neuroinflammation [78], and repairing damaged synaptic structures to improve neuroplasticity [79]. Additionally, it has been demonstrated to modulate HPA axis activity [49] and to restore balance to neuroendocrine disorders in conditions of oxidative stress [80]. Despite the fact that polysaccharide components are incapable of direct entry into the central nervous system, they have been demonstrated to enhance the intestinal microecological barrier by means of regulating gut microbiota composition, thereby reducing abnormal endotoxin secretion [81]. This indirectly influences central nervous function by means of metabolic pathways of the microbiota-gut-brain axis, establishing an intestinal immune barrier to block inflammatory signal transmission to the central nervous system. The combination of essential oils and polyphenolic components has been shown to produce a synergistic effect, enhancing antioxidant stress resistance and suppressing neuroinflammation [70]. This effect is further enhanced by the synergistic regulation of gut microbiota metabolism with saponins. This creates a bidirectional regulatory effect, characterized by the simultaneous protection of the local gut microbiota and the targeted repair of the central nervous system.

**Figure 3:**
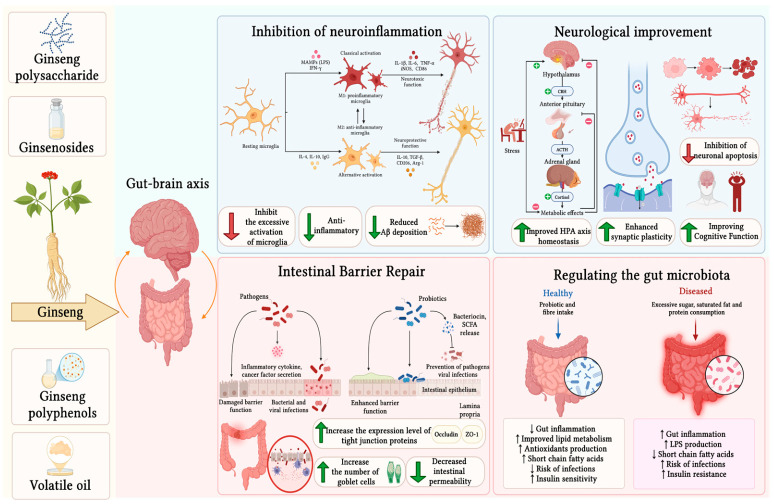
The active components of ginseng can target the gut-brain axis.

Overall, a substantial body of reliable preclinical evidence suggests that various bioactive components of ginseng exert targeted regulation on GBA through synergistic, multi-pathway, and multi-target mechanisms, with ginsenosides demonstrating the most comprehensive evidence across all regulatory pathways, followed by ginseng polysaccharides. These components achieve this regulation by acting on key processes, including maintenance of the intestinal barrier, modulation of the gut microbiota, suppression of neuroinflammation, clearance of oxidative stress, and restoration of neural function (Figure 3). These findings support the rationale for developing ginseng-based interventions targeting GBA; however, as most of the current data originate from preclinical models, further clinical validation is still required in the future.

## 5. Mechanism of Action of Ginseng in Targeted Regulation of GBA

### 5.1. Gut Microbiota Regulation

In recent years, the gut microbiota has garnered significant attention from the scientific community due to its close association with various health outcomes extending beyond the gastrointestinal system [82] to include immune function and metabolic health [83]. Consequently, the maintenance and restoration of microbial homeostasis in the gut is imperative for overall health. Prebiotic compounds are regarded as having significant value due to their distinctive characteristics. As food-grade substances, they are subject to degradation by microorganisms such as *Bifidobacteria* and *Lactobacillus*, resulting in the production of SCFAs [84]. Simultaneously, they selectively promote the growth and activity of beneficial bacteria in the gut, indirectly exerting positive effects on the host [85]. Li et al. [66] investigated the effects of ginseng polysaccharides (WGP) on gut microbiota diversity in mice with antibiotic-associated diarrhea. Results showed that compared to diarrhea-affected mice, WGP significantly altered gut microbiota composition and diversity. At the genus level, WGP increased the relative abundance of Lactobacillus, Lactococcus, and Streptococcus while decreasing that of Bacteroides. This finding suggests that WGP has the capacity to modify the composition and diversity of the gut microbiota in mice with antibiotic-associated diarrhea, thereby restoring the microbiota while balancing metabolic processes.

Simultaneously, ginseng exerts a prebiotic-like effect by promoting the growth of beneficial bacteria such as *Bifidobacteria* and *Lactobacillus* [86], which further enhances the production of SCFAs. SCFAs, primarily derived from the fermentation of dietary fiber by gut bacteria, have been demonstrated to play a pivotal role in the microbiota-gut-brain axis. This includes providing energy for the body [87], participating in immune regulation [88], and crossing the blood-brain barrier to interact with the vagus nerve, thereby influencing neural activity in the brain [89]. Yan et al. [90] used a D-galactose-induced MCI mouse model to confirm that CK repairs MCI-induced intestinal barrier dysfunction, enhances BBB integrity, increases gut microbiota diversity, modulates SCFAs levels, and alleviates MCI-induced dysbiosis.

However, it is important to note that observed changes in the gut microbiota alone are insufficient to establish causality, as correlation does not rule out the possibility that both the microbial changes and the improvement in treatment outcomes are secondary effects of ginseng’s other systemic actions. Among the studies included in this review, only a small fraction employed rigorous causal validation methods: fecal microbiota transplantation [45,91,92] demonstrated that the therapeutic effects could be replicated by microbiota transfer alone, while antibiotic depletion [63,90] eliminated the benefits of ginseng. Vagus nerve section studies [9,93] confirmed that the vagus nerve is a key signaling pathway, although these studies did not directly prove that the signals originate from microorganisms. Nevertheless, a synthesis of the included studies reveals that ginseng bioactive components consistently increase the relative abundance of beneficial genera such as *Lactobacillus*, *Bifidobacterium*, and *Akkermansia* across multiple pathological models. Currently, most studies remain at the level of correlation, which is one of the limitations in the gut-brain axis field; a series of targeted mechanistic validation experiments will still need to be conducted in the future.

### 5.2. Intestinal Barrier Protection

The intestinal barrier’s integrity is inextricably linked to intestinal homeostasis and health, as it prevents harmful substances from entering the bloodstream through the intestinal lumen [94]. Intestinal tight junction proteins, as pivotal molecules that form tight junctions between intestinal cells, play a crucial role in maintaining the integrity and permeability of the intestinal mucosal barrier [95]. Therefore, the expression levels of intestinal tight junction proteins serve as significant indicators for evaluating intestinal permeability and function. Su et al. [96] employed a middle cerebral artery occlusion/reperfusion (MCAO/R) mouse model to determine whether ginsenoside Rb1 could improve brain/lung/intestinal barrier damage via the proliferator-activated receptor-gamma (PPARγ) signaling pathway. Results demonstrated that GRb1 significantly mitigated multi-organ injury while increasing tight junction protein expression in the cerebral microvasculature, pulmonary vasculature, and intestinal epithelium. It reduced intestinal permeability by activating PPARγ, decreasing phosphorylated NF-κB levels, and suppressing pro-inflammatory cytokine production. This improvement in intestinal barrier dysfunction and maintenance of barrier homeostasis suggests a potential for therapeutic applications in related conditions. Ginseng polysaccharides, natural bioactive components extracted from ginseng, possess gut epithelial barrier-enhancing activity. A novel ginseng polysaccharide (GPH1) isolated from ginseng was demonstrated to improve hepatic lipid accumulation and inflammatory injury in obese mouse models while upregulating intestinal barrier tight junction protein expression [97].

Endotoxemia is a pathological state characterized by elevated levels of bacterial endotoxins in the bloodstream. Its primary component, lipopolysaccharide (LPS), activates various immune cells to release inflammatory mediators, increasing vascular permeability [98,99], exaggerating the inflammatory response, and exacerbating organ damage. Kang et al. [100] confirmed that Rg1 alleviates LPS-induced intestinal barrier damage by upregulating ZO-1, Occludin, and Claudin-1 expression. The magnitude of tight junction protein upregulation varies widely, primarily due to differences in extraction methods and treatment durations.

### 5.3. Immunity and Activation of Anti-Inflammatory Pathways

To understand the gut microbiota-mediated mechanism of Bifidobacteria-fermented red ginseng (fRG) against anxiety and depression, Han et al. [101] investigated the effects of red ginseng (RG), fRG, ginsenoside Rd, and protopanaxatriol on mouse anxiety, depression, colitis, and gut dysbiosis. They found that RG and fRG treatment significantly alleviated mouse anxiety and depression-like behaviors while inhibiting hippocampal NF-κB activation and NF-κB^+^/Iba1^+^ cell populations. Meanwhile, Rd and protopanaxatriol alleviated anxiety, depression, and colitis. These findings indicate that RG and its components Rd and protopanaxatriol alleviate anxiety, depression, and colitis by regulating NF-κB-mediated BDNF expression and gut dysbiosis. Concurrently, ginsenoside Rb1, a major active component of ginseng, exhibits potent anti-inflammatory activity. Findings by Gao et al. [102] confirm that Rb1 significantly alleviates LPS-induced acute kidney injury, primarily by modulating Toll-like receptor 4 (TLR4) dimerization and NF-κB/MAPK signaling pathways to exert anti-inflammatory effects in vitro and in vivo. Furthermore, Joh et al. [103] demonstrated that ginsenoside Rb1 and compound K inhibit the activation of interleukin-1 receptor-associated kinase-1 (IRAK-1), IKK-β, NF-κB, and MAP kinases. Their work confirmed that targeting IRAK-1 activation offers therapeutic potential for inflammatory diseases such as colitis. However, anti-inflammatory efficacy is more consistent in acute inflammation models, while results from chronic low-grade inflammation models remain partially contradictory.

### 5.4. Neuroendocrine and Neural Communication

#### 5.4.1. Regulate the Synthesis of Neurotransmitters

Neurotransmitters function as crucial mediators in the communication between the gastrointestinal system and the brain. The extensive microbial communities residing within the gastrointestinal tract play a pivotal role in immune regulation and metabolism [104]. Moreover, these communities have been shown to exert bidirectional regulatory effects on intestinal health [105] and central nervous system homeostasis [106,107]. Current research confirms that gut microbiota can exert bidirectional regulation by modulating the synthesis and release of neurotransmitters such as dopamine [108], norepinephrine [109], 5-HT [110], or gamma-aminobutyric acid (GABA) [111], exerting profound effects on the organism.

Dopamine has been linked to a variety of functions, including reward, motivation, and motor control. Maintaining sustained normal levels of dopamine bioavailability has been shown to have a positive impact on brain physiology and can play a role in the prevention of neurosystem-related diseases [112]. Concurrently, the gut microbiota possesses intrinsic enzymatic activity highly involved in dopamine metabolism. This activity promotes both dopamine synthesis and degradation [113]. Wang et al. [114] demonstrated that ginsenoside Rb1 mediates effects through 5-HT, noradrenergic, and dopaminergic systems, exhibiting significant antidepressant activity in behavioral tests, chronic animal models, and drug interactions.

5-HT exhibits multifaceted activity in major diseases associated with both gastrointestinal function [115] and neuroinflammation [116]. Chen et al. [117] established a neuropsychiatric model of morphine dependence, which is also intrinsically linked to gut microbiota dysbiosis. Results indicate that ginsenoside Rg1 significantly ameliorates morphine-induced gut microbiota dysbiosis. This effect is achieved by inhibiting tryptophan metabolism derived from the gut microbiota and reducing serotonin receptor levels in the serum, including 5-HT1B and 5-HT2A receptors. Xu et al. [118] demonstrated that ginsenoside Rg3 prevents decreases in progesterone, estradiol, and 5-HT levels in the prefrontal cortex and hippocampus, thereby improving anxiety- and depression-like behaviors in CUMS model mice.

GABA, an inhibitory neurotransmitter that plays a crucial regulatory role in the central nervous system, is involved in reducing neuronal excitability [119]. Consequently, GABA imbalance has been identified as a contributing factor to the onset and progression of neurological disorders. Recent findings support the hypothesis of a dynamic relationship between GABA levels, cerebral changes, and alterations in gut microbiota composition [120]. Shao et al. [121] discovered that ginsenosides Rg5 and Rk1 exert sedative and hypnotic effects by influencing the GABAergic system and 5-HT pathways. They increase the GABA/Glu ratio while upregulating 5-HT1A expression, with similar results observed for GABA and 5-HT in mouse cecal tissue.

#### 5.4.2. Interaction with the HPA Axis

The HPA axis is a critical element of the body’s stress response system. HPA dysfunction is associated with various mental and physical disorders, including depression [122], anxiety [123], and metabolic syndrome [124]. As a core component of the neuroendocrine system, the HPA axis directly mediates psychological and physical stress responses [125] and regulates numerous normal physiological processes. Simultaneously, the HPA axis facilitates direct communication with the gut microbiota. HPA axis activity has been demonstrated to alter gut microbiota composition and regulate intestinal permeability, thereby inducing chronic tissue inflammation [126]. Therefore, regulating the HPA axis holds significant promise for treating multiple diseases.

Ginseng has been demonstrated to modulate the HPA axis and exhibits significant biological activities, including anti-stress, neuroprotective, and immunomodulatory effects. Its precise regulation of HPA axis function is considered a key mechanism underpinning its role in modulating the gut-brain axis and treating diseases. Yang et al. [93] summarized the mechanisms by which ginsenoside Rg1 improves depressive-like behavior, including suppression of HPA axis hyperactivity, modulation of synaptic plasticity, and regulation of gut microbiota, highlighting Rg1′s potential in treating neuropsychiatric disorders. Findings by Shao et al. [127] demonstrated that ginsenoside Rh4 significantly mitigates neuronal and synaptic damage, alleviates depressive-like behaviors in mouse models of depression, and resolves HPA axis dysregulation. Concurrently, it improves gut microbiota composition, increases short-chain fatty acid levels, and inhibits the LPS/NLRP3/caspase-1/IL-1β signaling pathway, thereby exerting key therapeutic effects. Zhang et al. [128] demonstrated that red ginseng total saponins (RGTSF) significantly modulate HPA axis-related indicators while regulating gut-brain axis-related markers and improving spleen deficiency syndrome, highlighting ginseng’s potential in regulating the HPA axis and treating gut-related disorders. These effects are more pronounced in acute stress models, while greater interindividual variability is observed in chronic stress models.

#### 5.4.3. Vagus Neural-Mediated Signal Transduction

**Figure 4:**
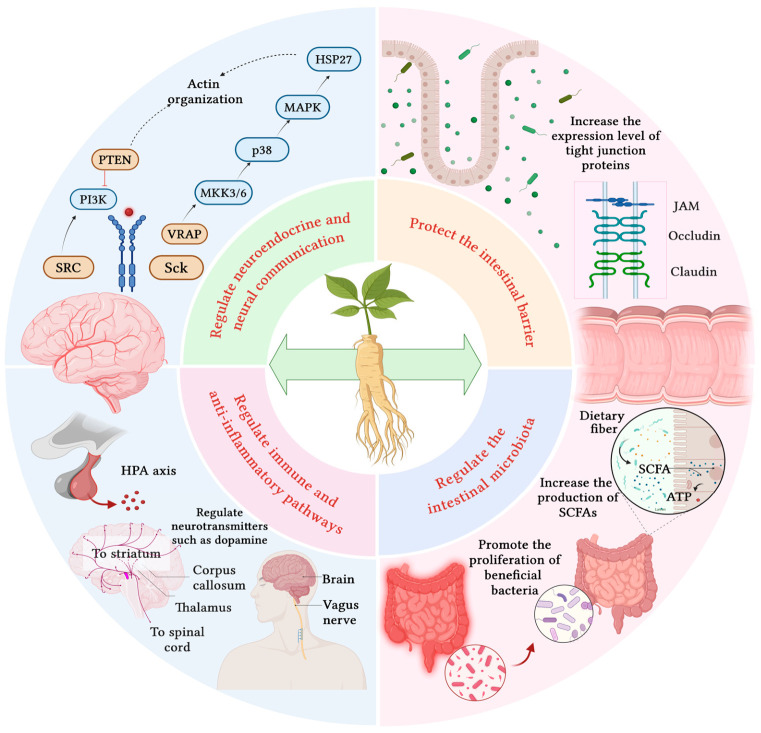
The mechanism by which ginseng targets and regulates the gut-brain axis.

The vagus nerve, a critical component of the parasympathetic nervous system, plays a pivotal role in facilitating communication between the gut and the brain [129]. Existing research has confirmed the antidepressant activity of ginsenosides. Rk3 has been shown to modulate tryptophan metabolism in the brain-gut axis by targeting tryptophan hydroxylase, thereby reshaping gut microbiota composition, mitigating intestinal barrier damage, and subsequently improving hypothalamic-pituitary-adrenal axis dysfunction in mice. This intervention has been shown to mitigate neuronal damage in the hippocampal and prefrontal cortical regions of mice, thereby ameliorating depressive-like behaviors [9]. Similarly, studies have confirmed Rg1′s anti-inflammatory, antioxidant, and neuroprotective activities. It exerts therapeutic effects in early-stage and mid-to-late-stage Alzheimer’s disease by repairing dendrites and axons and resolving inflammation associated with microglia and astrocytes. This primarily relies on its activation of the vagus nerve, thereby mediating gut-brain axis signaling pathways [93] (Figure 4).

The strongest causal evidence across all GBA regulatory mechanisms comes from rodent faecal microbiota transplantation and vagotomy studies, which have confirmed the central roles of gut microbiota remodelling and vagal nerve signalling in mediating ginseng’s effects. In vitro studies have also validated the molecular mechanisms of intestinal barrier protection and anti-inflammation. However, there have been no human studies that have directly measured the effects of ginseng on intestinal permeability or vagal nerve activity, and it is unclear how these preclinical mechanisms can be translated to humans. Additionally, the necessity of the vagus nerve pathway has only been verified in a limited number of disease models.

## 6. The Therapeutic Application of Ginseng Targeting the GBA in Diseases

### 6.1. Neurodegenerative Diseases

#### 6.1.1. Alzheimer’s Disease (AD)

Alzheimer’s disease is the most prevalent cause of dementia worldwide and is classified as a neurodegenerative disorder. This condition is characterized by the progressive degeneration of neurons, which is associated with the formation of β-amyloid plaques and neurofibrillary tangles [130]. The gut microbiota comprises complex microbial communities residing within the intestinal ecosystem. These microorganisms can participate in gut-brain axis activity, thereby influencing cognitive function and associated behaviors [131]. Growing evidence indicates that dysbiosis of the gut microbiota may compromise immune responses and promote inflammation, which could potentially impact the digestive or nervous systems [132]. This dysbiosis has been posited as a potential initiating factor in the onset and progression of AD. Ginsenosides, the active constituents of natural medicines, possess potent antioxidant properties. The mechanisms by which they exert their neuroprotective effects include the restoration of the gut-brain axis through modulation of the gut microbiota and brain neurotransmitters [133]. According to the findings of previous studies, the administration of Rg1 has been shown to alter the abundance of gut microbiota in mice, thereby improving AD, with Proteobacteria and Verrucomicrobia being key bacterial groups [134]. Lee et al. [91] found that oral red ginseng improved cognitive behavior in mice, reduced Aβ deposition and microglial hyperactivation, decreased blood-brain barrier permeability, and altered gut microbiota diversity by increasing Lactobacillus species. This demonstrates red ginseng’s ability to mitigate Alzheimer’s pathology via the gut-brain axis. Shin et al. [135] used a 5 × FAD transgenic mouse model of AD and found that the non-saponin fraction with rich polysaccharide (NFP) of red ginseng significantly reduced Aβ deposition in the hippocampi of model mice, alleviated neuroinflammation, neuronal loss, and mitochondrial dysfunction. It also improved mitochondrial defects in Aβ-treated HT22 cells and promoted cognitive recovery in AD mice, confirming that red ginseng polysaccharides exert therapeutic effects against AD by modulating the gut-brain axis.

#### 6.1.2. Parkinson’s Disease (PD)

PD is a neurodegenerative disorder characterized by the progressive loss of dopaminergic neurons in the substantia nigra of the midbrain and the subsequent abnormal aggregation of α-synuclein (α-syn) [136]. Recent studies have identified a potential role for the gut microbiota in the pathogenesis of PD through the gut-brain axis [137]. This suggests that α-syn accumulation in the gut may be transmitted to the brain through the vagus nerve, contributing to the development of PD. The inflammatory response and metabolite changes caused by the disorder of the gut microbiota are manifested as a sharp reduction in the content of short-chain fatty acids, which eventually aggravates nerve damage. Therefore, targeting the gut-brain axis holds significant promise for alleviating neuroinflammation and inhibiting α-syn pathological propagation. Currently, ginseng’s active components have been demonstrated to alleviate PD motor dysfunction by reshaping gut microbiota structure, enhancing intestinal barrier function, and suppressing inflammatory pathways [138]. Ginseng shows promising preclinical potential in treating neurodegenerative disorders. Research has confirmed that red ginseng modulates intestinal tight junctions and inflammation in the colon of a Parkinson’s disease mouse model, improves motor dysfunction, inhibits the disruption of tight junction proteins in the colon, reduces inflammatory factor levels, and prevents the accumulation of α-syn in the colon and substantia nigra. This indicates that red ginseng may influence the progression of Parkinson’s disease by regulating gut-related indicators, involving mechanisms related to the gut-brain axis [139].

### 6.2. Depression and Anxiety

Depressive disorders and anxiety disorders are currently the most prevalent mental illnesses worldwide, often occurring concurrently [140]. The underlying cause involves prolonged HPA axis stress, leading to abnormally elevated cortisol levels. These elevated cortisol levels are accompanied by varying degrees of hippocampal neuronal damage to the hippocampus in patients [141]. The bidirectionally regulated gut-brain axis has emerged as a novel therapeutic target for depression and anxiety. Research confirms that individuals with depression commonly exhibit gut microbiota dysbiosis and intestinal barrier dysfunction. This allows endotoxins to enter the bloodstream, activating peripheral immune cells to release pro-inflammatory factors. These signals are transmitted via the vagus nerve to the central nervous system, exacerbating neuroinflammation [142]. In a mouse model subjected to immobilization stress (IS) or transplanted with the feces of patients with ulcerative colitis and depression (UCDF), administration of RG and fermented red ginseng (fRG) was shown to regulate the microbiota-gut-brain axis. This intervention has been shown to ameliorate neuroinflammation and anxiety/depression-like behaviors in mice while concomitantly attenuating intestinal inflammation [45].

### 6.3. Metabolism-Related Diseases

#### 6.3.1. Obesity and Diabetes

Obesity and its associated type 2 diabetes mellitus (T2DM) are two inextricably linked metabolic disorders. According to statistical data, approximately 463 million individuals worldwide are affected. The underlying causes of these conditions are primarily attributed to the expansion of adipose tissue and systemic inflammatory responses triggered by genetic susceptibility or prolonged nutritional excess. This leads to a surge in pro-inflammatory cytokine levels, disrupting insulin signaling pathways [143]. Notably, T2DM is frequently accompanied by altered gut pathophysiology, manifesting as dysbiosis [144]. Concurrently, obesity disrupts the balance between beneficial probiotics and harmful pathogens within the body [145]. Thus, modulating this microbial imbalance presents a novel therapeutic avenue.

A growing body of research corroborates the notion that ginseng’s primary active components exert bidirectional regulation on the structure of gut microbiota, thereby promoting the proliferation of beneficial bacteria while impeding the growth of pathogenic microorganisms [146]. Wei et al. [147] investigated the effects of ginsenoside Rg5 on the gut microbiome of diabetic db/db mice. Data indicated that Rg5 treatment improved hyperglycemia, restored intestinal barrier function, alleviated inflammation associated with metabolic endotoxemia, and reversed dysbiosis in the colonic microbiota. Reversing gut dysbiosis and diabetes-associated metabolic disorders in a type 2 diabetes context, reducing intestinal permeability and subsequent systemic endotoxemia, contributes to maintaining normal gut-brain axis function. Administration of ginsenoside Rg1 significantly reduced blood glucose levels in high-fat diet and streptozotocin (STZ)-induced T2D rats, mitigating insulin resistance, oxidative stress, and inflammatory responses. It modulates gut microbiota composition by increasing the proportion of *Lachnospiraceae_NK4A136_group* and *Lachnoclostridium* while decreasing Lactobacillus content, thereby improving gut microbial composition and exerting anti-diabetic activity [148]. Hyperlipidemia is also one of the hallmarks of type 2 diabetes. Gamma-aminobutyric acid-fructose-glucose (GABAFG), a Maillard reaction product of ginseng, increased *Akkermansia* and decreased *Romboutsia* abundance in a high-fat diet/streptozotocin (HFD/STZ)-induced T2DM mouse model of hyperglycemia and hyperlipidemia. This reshaped the gut microbiota and reduced serum glycerophospholipid metabolism, thereby alleviating T2DM-induced dyslipidemia [149]. Han et al. [150] found that ginseng peptides (GP) significantly reduced fasting blood glucose levels, improved insulin resistance (IR), and corrected lipid metabolism disorders in T2DM mice. while also regulating gut microbiota dysbiosis and short-chain fatty acid metabolism. GP activates the IRS-1/PI3K/Akt and AMPK signaling pathways to modulate glycogen synthesis, gluconeogenesis, and glucose transport, and improves IR by inhibiting mitochondrial apoptosis signaling pathways. Polysaccharides, as one of the primary active components in ginseng, have been found to possess hypoglycemic and hypolipidemic activities. Investigations into the effects of rhamnogalacturonan-I-enriched pectin (GPS-1), abundant in ginseng, on lipid metabolism in T2DM rats revealed that GPS-1 modulates gut microbiota composition, increases short-chain fatty acid levels, and improves lipid metabolism by activating the AMP protein kinase pathway [151].

#### 6.3.2. Non-Alcoholic Fatty Liver Disease (NAFLD)

The pathological hallmark of NAFLD is excessive accumulation of lipids in the liver without significant alcohol consumption. Its progression can range from simple steatosis to non-alcoholic Steatohepatitis (NASH), fibrosis, and ultimately cirrhosis [152]. The pathogenesis of NAFLD is primarily driven by insulin resistance, dyslipidemia, and chronic inflammation. Studies indicate that high-fat diets induce gut microbiota dysbiosis, characterized by reduced abundance of beneficial bacterial genera [153,154]. This dysbiosis compromises the intestinal barrier, allowing microbial byproducts and endotoxins to translocate to the liver via the portal vein. Within the liver, these substances trigger mitochondrial dysfunction, oxidative stress, and lipogenesis [155,156].

Ginsenosides exhibit structural similarity to steroid hormones [157]. Research indicates that ginsenoside extract (GE) exerts a significant therapeutic effect on symptoms of NAFLD induced by a high-fat diet (HFD), exhibiting a dose-dependent response. In addition to its efficacy in alleviating NAFLD symptoms, GE has been observed to promote gut microbiota balance, reduce intestinal permeability, and mitigate metabolic endotoxemia [158]. Shi et al. [92] similarly identified ginsenoside Rg5-mediated gut-microbiome axis activity in an HFD-induced NAFLD model to exert therapeutic effects against NAFLD. Rg5 intervention altered gut microbiota composition, promoting increases in beneficial bacteria such as *Bacteroides* and *Akkermansia* while reducing the relative abundance of harmful bacteria. Similar outcomes were observed in fecal microbiota transplantation (FMT) experiments, further confirming Rg5′s ability to mitigate NAFLD activity in mice by actively participating in gut microbiota restoration via FMT.

### 6.4. Digestive System Diseases

#### 6.4.1. Inflammatory Bowel Disease (IBD)

Inflammatory bowel disease (IBD) is a group of chronic inflammatory conditions primarily comprising Crohn’s disease (CD) and ulcerative colitis (UC). The etiology of the condition under investigation may be attributed to a combination of genetic predisposition, immune dysregulation, environmental factors, and gut microbiota dysbiosis [159]. Typically, patients suffering from IBD exhibit a significant imbalance in the composition of their gut microbiota. Concurrently, the disruption of microbial metabolic functions leads to reduced SCFA synthesis, impaired tryptophan metabolism, and abnormal bile acid conversion. These alterations further compromise intestinal barrier integrity and exacerbate inflammation [160]. Consequently, modulating gut microbiota has emerged as a novel therapeutic strategy for IBD. Ginseng active components, such as ginsenosides, have demonstrated preclinical therapeutic effects through multi-targeted interventions in microbiota-host interactions [161]. Research indicates that the primary metabolites of American ginseng following intestinal microbial conversion are CK and ginsenoside Rg3. These metabolites significantly alleviate chemically induced colitis and abdominal pain by suppressing pro-inflammatory cytokine expression, demonstrating that ginseng’s gut microbial metabolites possess promising preclinical therapeutic activity against inflammatory bowel disease [162].

#### 6.4.2. Ulcerative Colitis (UC)

**Figure 5:**
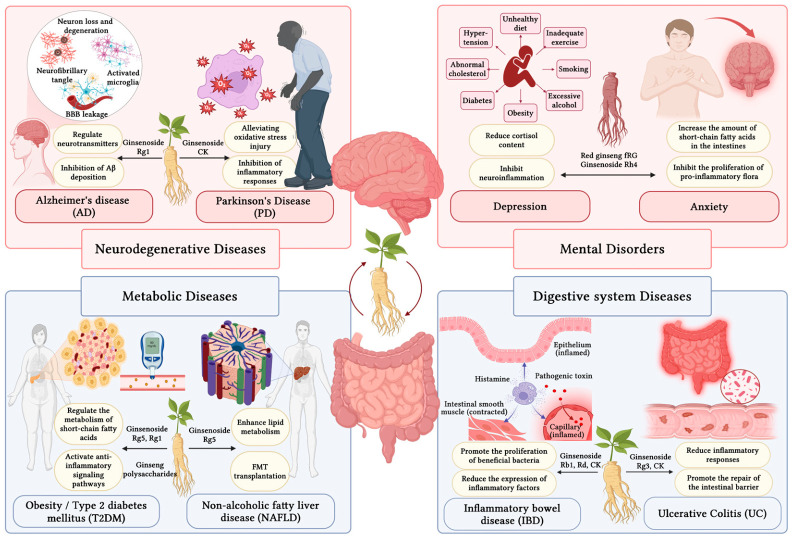
The active components of ginseng target the regulation of GBA in the treatment of diseases.

UC is a chronic, non-specific inflammatory bowel disease. Its pathogenesis has been linked to genetic factors [163], immune dysregulation [164], gut microbiota imbalance [165], and environmental influences [166]. At present, the modulation of the gut microbiota has emerged as a highly promising therapeutic strategy for ulcerative colitis. Ginseng, a time-honored herbal remedy, is characterized by its active constituents, which exhibit a wide array of biological functions. Liu et al. [167] established a UC model using dextran sulfate sodium (DSS) and found that ginsenoside Rg3 administration suppressed NLRP3 inflammasome activation, pyroptosis, and apoptosis while partially restoring gut microbiota abundance at phylum and genus levels in UC mice. Meanwhile, Shin et al. [45] established a UC-associated anxiety and depression model by exposing mice to restraint stress or transplanting fecal matter from UC and depression patients, and found that ginsenoside Rd and CK suppressed anxiety-depression-like behaviors while partially restoring gut microbiota fluctuations induced by fecal transplants from UC and depression patients. This suggests they may alleviate colitis by modulating the microbiota-gut-brain axis (Figure 5).

In terms of therapeutic applications, there is the most extensive preclinical evidence for neurodegenerative diseases and mood disorders, with consistent findings across multiple independent rodent models. Metabolic and gastrointestinal diseases also show promising preclinical results. However, almost all studies are currently limited to animal models, with only a few indications having been investigated in small-scale observational studies and pilot trials. To date, no large-scale randomised controlled trials have evaluated the efficacy of ginseng components targeting the GBA in any human disease. Additionally, critical clinical translation gaps remain, including insufficient long-term safety data, a lack of standardized dosing regimens across different ginseng preparations, and significant interindividual variability in response driven by gut microbiota composition differences.

## 7. Future Perspectives

Looking ahead, the application of ginseng’s active components in regulating the gut-brain axis and treating related diseases requires targeted translational research. In the field of nanodelivery systems, three testable directions should be prioritized to address the poor oral bioavailability of ginsenosides: colon-targeted carriers that protect components from upper gastrointestinal degradation and release them locally to modulate gut microbiota and barrier function; dual-targeted nanocarriers that cross both the intestinal and blood-brain barriers for simultaneous peripheral and central regulation; and stimuli-responsive systems that release payloads in response to colonic low pH or inflammatory mediators. Such synergistic effects offer more effective interventions for gut-brain axis-related disorders like irritable bowel syndrome and depression.

Regarding synergistic strategies, the combination of ginseng active components and probiotics leverages functional complementarity: ginsenosides promote the proliferation of beneficial bacteria such as *Lactobacillus* and *Bifidobacterium*, while short-chain fatty acids enhance the anti-inflammatory and neuroprotective activities of ginseng constituents, forming a virtuous “ginseng-microbiota-gut-brain” interaction cycle. Synergistic combinations with other herbs can target multi-pathway interactions. For instance, pairing ginseng with herbs like Bupleurum and Astragalus modulates the gut microbiome-immune-HPA axis network, enhancing comprehensive intervention for disorders stemming from gut-brain axis dysregulation.

In personalized medicine, integrated microbiome and metabolomics stratification will guide tailored applications of ginseng preparations. By analyzing patient gut microbiota composition and metabolic characteristics through metagenomic sequencing and pharmacometabolomics, the association between specific microbial structures and the metabolic efficiency of ginseng active components can be clarified. This enables the customization of formulations containing specific ginsenosides and metabolomics-guided personalized dosing, achieving precise matching between “microbiome characteristics-ginseng components-therapeutic response.” This approach will advance ginseng active components from empirical application toward precision treatment in multifactorial diseases related to the gut-brain axis.

Notably, several critical clinical translation challenges remain unaddressed in current research, which represent key priorities for future investigation. First, high-quality human clinical evidence specifically investigating the gut-brain axis-mediated effects of ginseng is extremely limited, and most existing human studies have not measured GBA-specific biomarkers to confirm the underlying mechanisms. Second, there is extensive significant variability in the chemical composition and biological efficacy of different ginseng preparations, including red ginseng, fermented ginseng, American ginseng, purified ginsenoside extracts, ginseng polysaccharides, and compound K, which complicates direct comparison of study results and standardization of clinical regimens. This variability stems from species differences, cultivation conditions, processing techniques, and extraction methods, leading to inconsistent findings across studies. Third, the poor oral bioavailability of ginsenosides and the lack of comprehensive pharmacokinetic data in humans hinder the rational design of clinical doses. The oral bioavailability of most major ginsenosides is less than 5% in humans, and they act primarily as prodrugs requiring gut bacterial metabolism to form bioactive metabolites. Critically, nearly all preclinical studies use doses 10–100 times higher than physiologically achievable levels in humans, limiting direct clinical extrapolation. Fourth, well-defined dose-response relationships and long-term safety data for chronic ginseng supplementation in different patient populations are still insufficient.

Although these preclinical findings are encouraging, there remains a gap in translating them into clinical practice. For future research, priority should be given to large-scale randomized controlled trials using chemically standardized ginseng preparations, with a focus on detecting GBA-specific biomarkers to optimize clinically relevant dosages and assess safety. Development of colon-targeted delivery systems to improve ginsenoside bioavailability is also urgently needed. This is crucial for advancing progress in this field.

## 8. Conclusions

In summary, multiple active components in ginseng demonstrate significant multi-target effects and therapeutic versatility in regulating the GBA. Evidence indicates that ginseng exerts multi-target regulatory effects on the GBA, supporting their promising preclinical therapeutic potential in gut-brain axis-related multifactorial diseases by acting on multiple nodes, including the gut microbiome, intestinal barrier integrity, immune regulatory networks, and central nervous system signaling pathways. However, it is critical to emphasize that nearly all supporting evidence to date derives exclusively from preclinical animal and in vitro studies. Current research requires enhanced translational medicine exploration; most evidence originates from animal models, necessitating resolution of issues such as dose-response relationships in humans, long-term safety, oral bioavailability limitations, and interindividual variability in gut microbiota-mediated metabolism. Future research should prioritize large-scale, well-designed randomized controlled trials using standardized ginseng preparations, combined with multi-omics approaches to bridge the gap between traditional herbal knowledge and modern clinical practice. Only through such an approach can the therapeutic value of ginseng’s active components in treating gut-brain axis-related disorders be fully realized, offering novel intervention strategies for multifactorial complex diseases that combine traditional foundations with scientific evidence.

## Data Availability

No new data were created or analyzed in this study.

## Abbreviations

The following abbreviations are used in this manuscript:

AD	Alzheimer’s disease
AAD	Antibiotic-associated diarrhea
ANS	Autonomic nervous system
α-syn	α-synuclein
BBB	Blood-brain barrier
CD	Crohn’s disease
CNS	Central nervous system
DSS	Dextran sulfate sodium
ENS	Enteric nervous system
fRG	Bifidobacteria-fermented red ginseng
FMT	Fecal microbiota transplantation
GBA	Gut-brain axis
GABA	Gamma-aminobutyric acid
GTSs	Ginseng total saponins
GP4	4.7-kDa ginseng-derived polysaccharide
GPN	Ginseng neutral polysaccharides
GRb1	Ginsenoside Rb1
GPH1	Ginseng polysaccharide
GP	Ginseng peptides
GE	Ginsenoside extract
GPS-1	rhamnogalacturonan-I enriched pectin
HPA axis	Hypothalamic-pituitary-adrenal cortex axis
HFD	High-fat diet
HFD/STZ	High-fat diet/streptozotocin
IRAK-1	Interleukin-1 receptor-associated kinase-1
IS	Immobilization stress
IR	Insulin resistance
IBD	Inflammatory bowel disease
LPS	Lipopolysaccharide
MCAO/R	Middle cerebral artery occlusion/reperfusion
NFP	Non-saponin fraction with rich polysaccharide
NAFLD	Non-alcoholic fatty liver disease
NASH	Non-alcoholic Steatohepatitis
PD	Parkinson’s disease
PPARγ	Proliferator-activated receptor-gamma
PTSD	Post-traumatic stress disorder
RG	Red ginseng
STZ	Streptozotocin
SCFAs	Short-chain fatty acids
TNF-α	Tumor Necrosis Factor-alpha
TLR4	Toll-like receptor 4
T2DM	Type 2 diabetes mellitus
UDCA	Ursodeoxycholic acid
UCDF	the feces of patients with ulcerative colitis and depression
UC	Ulcerative colitis
WGP	ginseng polysaccharides
5-HT	5-hydroxytryptamine
